# A focused simulation-based optimization of print time and material usage with respect to orientation, layer height and support settings for multi-pathological anatomical models in inverted vat photopolymerization 3D printing

**DOI:** 10.1186/s41205-021-00112-w

**Published:** 2021-08-26

**Authors:** Prashanth Ravi, Victoria C. P. Chen

**Affiliations:** 1 Department of Radiology, University of Cincinnati College of Medicine, 234 Goodman St, Cincinnati, OH 45219 USA; 2 Department of Industrial, Manufacturing and Systems Engineering, University of Texas at Arlington, 500 West First St, Arlington, TX 76019 USA

**Keywords:** Model orientation, Layer height, Medical 3D Printing, Print time, Material usage, Optimization, Inverted vat photopolymerization, Anatomical Models

## Abstract

**Background:**

3D printing of anatomical models requires multi-factorial decision making for optimal model manufacturing. Due to the complex nature of the printing process, there are frequently multiple potentialities based on the desired end goal. The task of identifying the most optimal combination of print control variables is inherently subjective and rests on sound operator intuition. This study investigates the effect of orientation, layer and support settings on print time and material usage. This study also presents a quantitative optimization framework to jointly optimize print time and material usage as a function of those settings for multi-pathological anatomical models.

**Methods:**

Seven anatomical models representing different anatomical regions (cardiovascular, abdominal, neurological and maxillofacial) were selected for this study. A reference cube was also included in the simulations. Using PreForm print preparation software the print time and material usage was simulated for each model across 4 orientations, 2 layer heights, 2 support densities and 2 support tip sizes. A 90–10 weighted optimization was performed to identify the 5 most optimal treatment combinations that resulted in the lowest print time (90% weight) and material usage (10% weight) for each model.

**Results:**

The 0.1 mm layer height was uniformly the most optimal setting across all models. Layer height had the largest effect on print time. Orientation had a complex effect on both print time and material usage in certain models. The support density and the support tip size settings were found to have a relatively minor effect on both print time and material usage. Hollow models had a larger support volume fraction compared to solid models.

**Conclusions:**

The quantitative optimization framework identified the 5 most optimal treatment combinations for each model using a 90–10 weighting for print time and material usage. The presented optimization framework could be adapted based on the individual circumstance of each 3D printing lab and/or to potentially incorporate additional response variables of interest.

## Background

Three-dimensional printing is increasingly employed to manufacture patient-specific anatomical models, medical devices and simulation models for training [1, 2]. There are several categories of 3D printing technology. The inverted Vat Photopolymerization (VP) technology is being used more frequently within academic medical centers due to its relative affordability as hospitals invest in point-of-care 3D printing facilities. There are two main types of VP technology including top-down laser-based VP generally used in industrial machines with large build volumes, and bottom-up laser based VP with relatively smaller machines also frequently referred to by the terminology desktop stereolithography (SLA) [3]. Bottom-up LCD/DLP VP printers are also available in the desktop segment. The technology employed in this paper is the bottom-up laser-based VP used in the Formlabs 3D printers [4]. Given that 3D printing is a complex multi-factorial process, there are often trade-offs between desired end goals. For instance, model accuracy and surface quality can be improved by reducing the layer height and/or re-orienting the model, but frequently at the expense of increased print time and cost [5–7]. Furthermore, the same control variables used across models can yield different output responses highlighting the model-specific nature of the manufacturing process [8]. The organic and complex model geometries present in anatomical models offer further unique challenges in such decision making. Advanced technologies such as machine learning and novel support generation strategies have been employed in 3D printing to optimize response variables such as energy consumption and material waste [9–11].

Kamio et al. found that an increase in layer height reduced the print time and material cost for a mandibular model without significant reduction in geometric accuracy [12]. Rubayo et al. found a significant difference in print time between dental surgical templates manufactured vertically vs. horizontally [13]. Some research groups have studied the accuracy of medical models and found the accuracy to be affected by multiple factors such as orientation, layer height and the cure settings [4, 14–17]. However, a focused investigation of the effect of different model geometries and critical print variables on important response variables is lacking in the medical 3D printing literature. Furthermore, the decision-making scheme surrounding model orientation and choice of print settings to achieve desired end goals remains unclear for the 3D printing of anatomical models and implants. For instance, optimizing for print time is crucial when a 3D printed model is needed for an emergency or trauma case.

The goal of this research was to investigate the effect of model orientation, layer height, support density and support tip size on print time and material usage in VP 3D printing based on print simulation for 7 anatomical models. We propose an adaptable optimization framework to jointly optimize both print time and material usage which could be tailored based on the availability of resources at the 3D printing laboratory. The framework enables the identification of optimal combinations of control variables using a quantitative approach and could serve as a useful guide to the 3D printer operator. The optimization scheme could also be further extended to include other response variables of interest based on the desired end goal.

## Methods

### Image segmentation and model STL creation

**Fig. 1:**
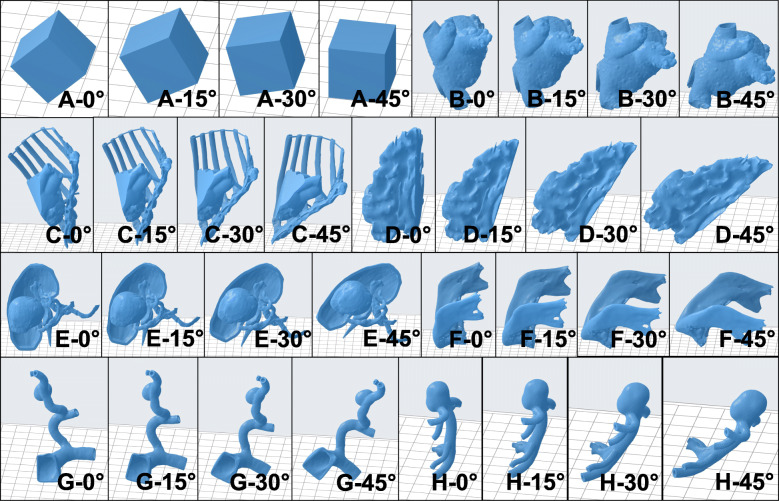
The four orientations of all 8 models simulated in this study shown without support structures. Starting from the top-left, 0 to 45-degree orientations of the (**A**) cube model, (**B**) left atrial appendage (LAA) model, (**C**) minimally invasive coronary artery bypass (MICAB) model, (**D**) low grade glioma (LGG) model, (**E**) renal cell carcinoma (RC) model, (**F**) mandibular osteonecrosis (MO) model, (**G**) hepatic pseudoaneurysm (HPA) model and (**H**) basilar tip aneurysm (BTA) model. Note that the models are not to scale and the corresponding STL volumes (**A**-**H**) are provided in Table 1

Image sets across anatomical regions and pathologies were selected for this study as part of the clinical 3D printing service line at the University of Cincinnati Department of Radiology. These included models for: left atrial appendage (LAA) occluder device sizing, minimally invasive coronary artery bypass (MICAB) surgery, low grade glioma (LGG) excision, renal cell carcinoma (RC) surgery, mandibular osteonecrosis (MO) resection, hepatic pseudoaneurysm (HPA) surgery and basilar tip aneurysm (BTA) clipping (Fig. 1). The models represent a range of volumes from ~ 2 cc to ~ 100 cc (Table 1). All data was completely de-identified and the research was submitted to the hospital research ethics board, and the study was considered exempt from further review, based on the fact that no data in this study could be used to identify any human being. The relevant anatomical regions were segmented from CT and MRI derived DICOM images using Materialise Mimics InPrint 3.0 (Materialise, Leuven, Belgium). The thresholding presets for Bone (226 to 3071 HU), Blood Vessel (200 to 3071 HU) and Kidneys (20 to 135 HU) within the software were used for CT images. The segmentation of relevant anatomical regions from MRI were performed using semi-automatic and manual segmentation methods. The LAA, HPA and BTA models were generated by constructing a 1.00–1.50 mm wall around the segmented blood pool. All other models including the reference cube were fully solid. The STL files were post-processed in Materialise 3-matic 15.0 (Materialise, Leuven, Belgium) where they were fixed, smoothed and wrapped. The smoothing factor was set to 0.90. Spike removal was performed on the RC and MO models with the spike size and smallest detail both set to 0.25 mm. The LAA model consisted of the appendage, atrium and pulmonary veins; the MICAB model consisted of the heart, left anterior descending artery, left interior mammary artery, sternum and ribs; the LGG model consisted of the glioma and white matter; the RC model consisted of the tumor, kidney shell and arterial as well as venous branches; the MO model consisted of the lower mandible; the HPA and BTA models consisted of the vessel wall and corresponding aneurysms (Fig. 1).

**Table 1:** The 8 models investigated in this study with their corresponding STL volumes and internal geometry type

Label	Model	Type	STL Volume (cc)
A	Cube (reference)	Solid	8.00
B	Left atrial appendage (LAA)	Hollow	30.25
C	Minimally invasive coronary artery bypass (MICAB)	Solid	100.33
D	Low grade glioma (LGG)	Solid	86.14
E	Renal cell carcinoma (RC)	Solid	73.92
F	Mandibular osteonecrosis (MO)	Solid	67.55
G	Hepatic pseudoaneurysm (HPA)	Hollow	3.47
H	Basilar tip aneurysm (BTA)	Hollow	1.98

### Study design and print simulation in PreForm

The study consisted of 8 models spanning a range of volumes, anatomical regions/pathologies and included 3 hollow models (Table 1). A 20 mm × 20 mm × 20 mm cube was included as reference geometry. The Print Time (PT) and Material Usage (MU) for each model was simulated in PreForm 3.9.0 (Formlabs, Somerville, MA, USA) with the resin set to Clear and printer set to the Form 3 (Formlabs, Somerville, MA, USA). Clear resin was used since it is the material of choice for most anatomical models at our lab because it allows the visualization of internal features. The PT and MU values included the model, support structure as well as raft for each print simulation. Four model orientations (ORN) – 0, 15, 30, 45 degrees, two layer heights (LH) – 0.05, 0.10 mm, two support densities (SD) – 0.80, 1.00 and two support tip sizes (STS) – 0.4, 0.5 mm were tested for a total of 32 treatment combinations (4 ORN × 2 LH × 2 SD × 2 STS) per model. The 4 orientation levels were chosen to represent common model orientation choices in inverted VP 3D printing. The 2 layer heights are most commonly used for 3D printing anatomical models. The lowest 0.025 mm layer height is typically only used when the highest accuracy is needed such as for surgical guides or implants that form part of an assembly. The 2 support densities and 2 support tip sizes represent the commonly used levels that satisfy “printability” in PreForm while resulting in good surface quality (larger tip sizes result in reduced surface quality). Each of the 32 treatments per model was simulated only once since there is no expected random error associated with this type of simulation. The 0-degree orientation was defined for each model as the most vertical orientation. The models were then rotated about the X-axis (which runs front-to-back in PreForm) in 15-degree increments (Fig. 1). The Z-height therefore reduced with increasing ORN for each model. Each model was placed in the center of the build plate for consistency between simulations. Support structures were generated both inside and outside the model since during actual 3D printing these internal supports would be needed for hollow models.

### Data analysis and optimization

The PT and MU were averaged by the 4 control variables – ORN, LH, SD and STS to assess their effect on PT and MU. A weighted optimization of PT and MU was performed with a weightage of 90% to PT and 10% to MU with the goal to identify the combination of control variables that resulted in the minimal weight. PT was given 90% weight since it is a more important parameter, particularly when 3D printing models for urgent procedures and for smaller 3D printing laboratories seeking to maximize their throughput. The raw optimization equations below were fed into Microsoft Excel (Microsoft, Redmond, WA, USA) for computation. The 5 most optimal solutions for each of the 8 models were further analyzed. The optimization used the following approach:

1 $$ {PT}_{Center}=\frac{\mathit{\operatorname{Max}}(PT)+\mathit{\operatorname{Min}}(PT)}{2} $$

*PT*_*Center*_ is an intermediate variable to store the average of the endpoints of the PT dataset for a model.

2 $$ {PT}_{Bottom}=\frac{\mathit{\operatorname{Max}}(PT)-\mathit{\operatorname{Min}}(PT)}{2} $$

*PT*_*Bottom*_ is an intermediate variable to store half the difference between endpoints of the PT dataset for a model.

3 $$ {PT}_{Normalized}=\frac{PT-{PT}_{Center}}{PT_{Bottom}} $$

*PT*_*Normalized*_ is an intermediate variable to store the normalized PT of a single PT data point for a model. *MU*_*Normalized*_ was calculated similarly using Eq. 1, Eq. 2 and Eq. 3 but using the corresponding MU values.

4 $$ W=0.90\times {PT}_{Normalized}+0.10\times {MU}_{Normalized} $$

W represents the weight of the final optimization. The lower the W, the more optimal the combination of input variables which yield the corresponding PT and MU.

## Results

The average PT ranged from ~ 2 to ~ 14.5 h and the average MU ranged from ~ 5 to ~ 130 mL for the 8 models (Table 2). The average support volume as a fraction of total volume ranged from ~ 15 to ~ 60%. This fraction ranged from ~ 15–23% for Solid models and ~ 47–60% for Hollow models. The range of coefficient of variation (std. dev./avg.) was higher for PT (0.28–0.32) than MU (0.02–0.11) across the 8 models.

**Table 2:** Summary of the Print Time (PT), Material Usage (MU) and average support to total volume ratio for all 8 models

Model	Avg. PT ± Std. Dev. (min)	Avg. MU ± Std. Dev. (mL)	Avg. Support/Total Volume (%)
Cube	109.8 ± 33.3	10.3 ± 0.4	22.3
LAA	680 ± 188.6	56.5 ± 3.5	46.5
MICAB	876.1 ± 252.5	129.5 ± 3.3	22.5
LGG	565.3 ± 156.8	100.7 ± 5.0	14.5
RC	678.1 ± 198.0	94.4 ± 1.8	21.7
MO	610.2 ± 169.8	87.8 ± 2.6	23.1
HPA	272.7 ± 98.2	7.3 ± 0.8	52.5
BTA	165.9 ± 52.5	5 ± 0.4	60.4

### A – cube

**Fig. 2:**
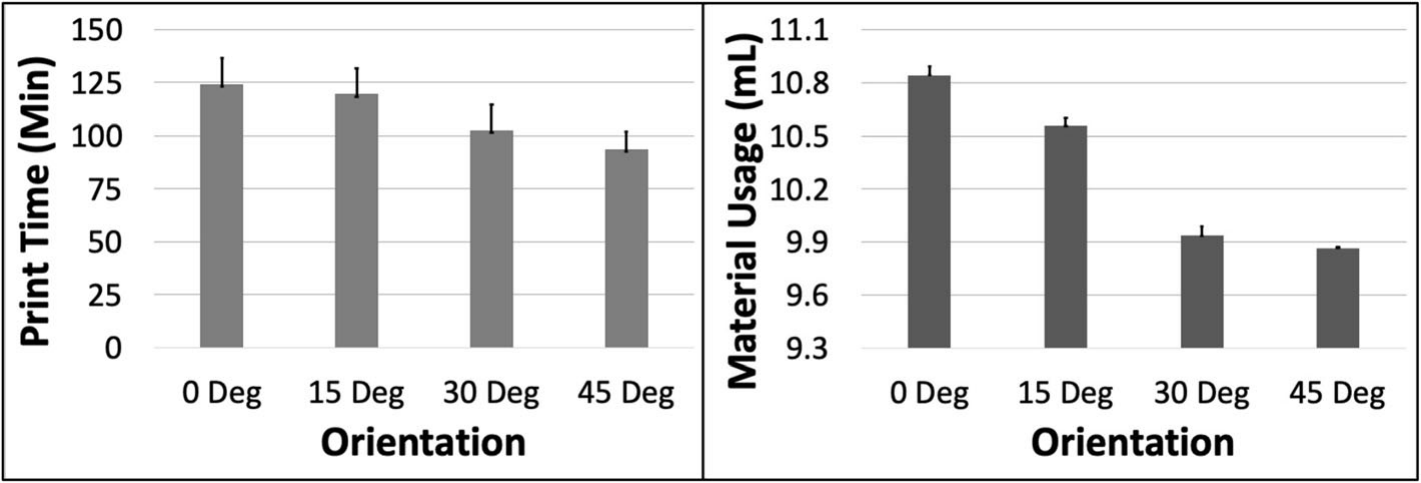
The response variables Print Time (PT) and Material Usage (MU) plotted against Orientation (ORN) for the Cube model. Sample size is 8 samples for each PT and MU average and the associated error bar

**Fig. 3:**
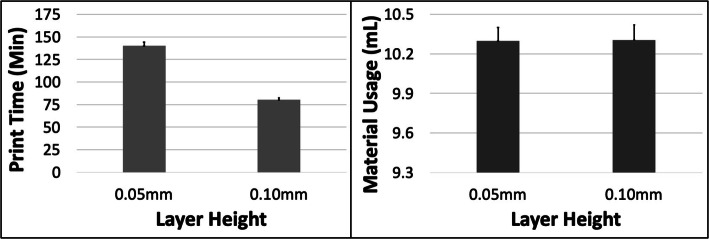
The response variables Print Time (PT) and Material Usage (MU) plotted against Layer Height (LH) for the Cube model. Sample size is 16 samples for each PT and MU average and the associated error bar

The PT and MU both reduced with increasing ORN for the Cube model (Fig. 2). The reduction in PT was more substantial (~ 25%) compared to MU (~ 10%) between the 0 and 45 deg levels. An increase in LH had the most impact on PT (~ 43% reduction) but very little effect on the MU (< 0.1%) (Fig. 3). The 5 most optimal (weighted) treatment combinations for the Cube model all consisted of the 30 and 45 deg ORNs and 0.1 mm LH (Table 3). These optimal combinations consisted of both SD and STS levels due to their relatively minor effect on both PT and MU.

**Table 3:** The 5 most weighted (W) optimal treatment combinations for the Cube model

ORN (Deg)	LH (mm)	SD	STS (mm)	MU (mL)	PT (min)	W
30	0.1	0.8	0.5	9.79	67	−1.0000
30	0.1	1	0.4	9.92	68	−0.9586
30	0.1	1	0.5	9.97	69	−0.9304
45	0.1	0.8	0.4	9.87	71	−0.9075
45	0.1	1	0.4	9.87	71	−0.9075

### B – left atrial appendage (LAA)

**Fig. 4:**
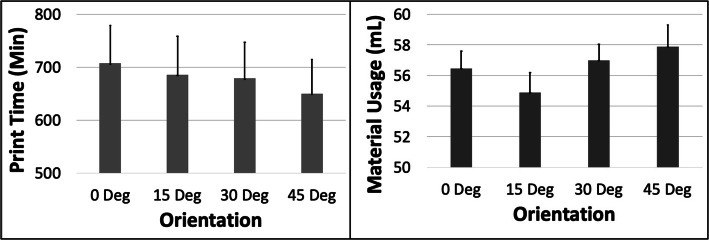
The response variables Print Time (PT) and Material Usage (MU) plotted against Orientation (ORN) for the LAA model. Sample size is 8 samples for each PT and MU average and the associated error bar

**Fig. 5:**
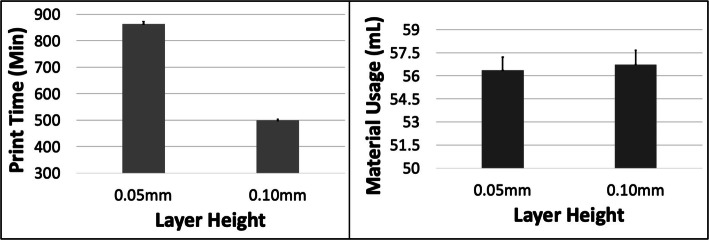
The response variables Print Time (PT) and Material Usage (MU) plotted against Layer Height (LH) for the LAA model. Sample size is 16 samples for each PT and MU average and the associated error bar

**Fig. 6:**
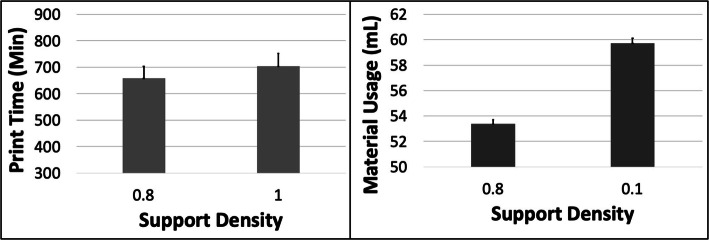
The response variables Print Time (PT) and Material Usage (MU) plotted against Support Density (SD) for the LAA model. Sample size is 16 samples for each PT and MU average and the associated error bar

The PT reduced with increasing ORN for the LAA model but the pattern was more complex for MU with an initial reduction followed by a subsequent rise (Fig. 4). The reduction in PT was more substantial (~ 8%) between the 0 and 45 deg levels compared to the increase in MU (~ 5%) between the 15 deg and 45 deg levels. An increase in LH caused a ~ 42% reduction in PT but barely any change (< 1%) in MU (Fig. 5). An increase in SD caused an increase in both PT of ~ 7% and in MU of ~ 12% (Fig. 6). The 5 most optimal (weighted) treatment combinations consist of the 0.1 mm LH and 0.80 SD with mostly larger ORNs (Table 4), and both levels of STS due to the minor effect of this control variable.

**Table 4:** The 5 most weighted (W) optimal treatment combinations for the LAA model

ORN (Deg)	LH (mm)	SD	STS (mm)	MU (mL)	PT (min)	W
45	0.1	0.8	0.5	53.21	454	−0.9639
45	0.1	0.8	0.4	54.05	456	−0.9409
15	0.1	0.8	0.5	51.24	472	−0.9314
15	0.1	0.8	0.4	51.35	476	−0.9141
30	0.1	0.8	0.5	54.47	480	−0.8417

### C – minimally invasive coronary artery bypass (MICAB)

**Fig. 7:**
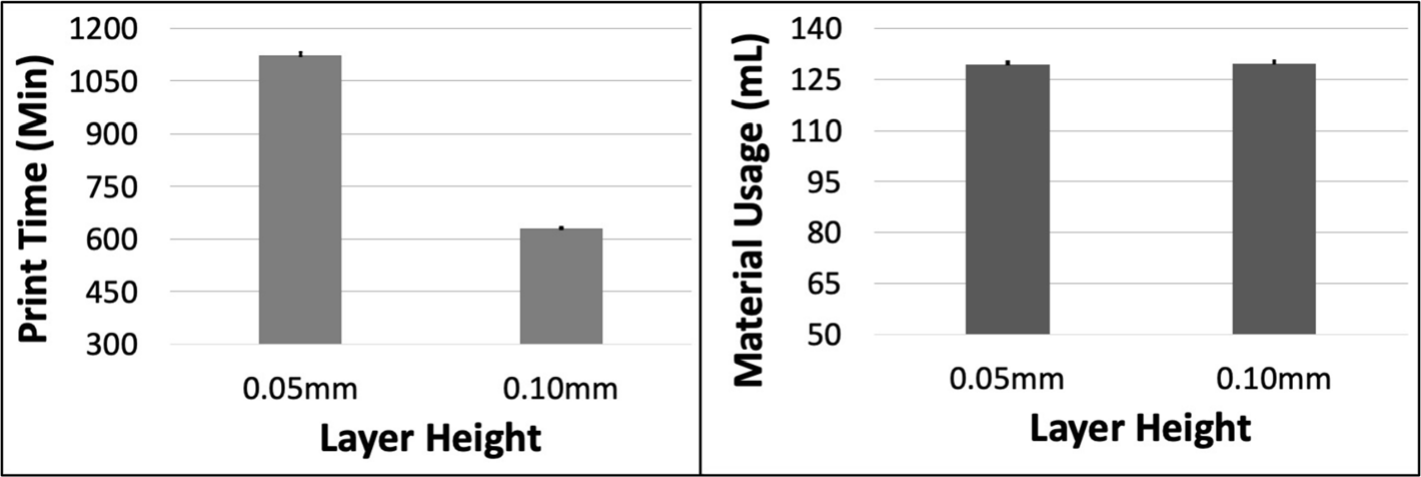
The response variables Print Time (PT) and Material Usage (MU) plotted against Layer Height (LH) for the MICAB model. Sample size is 16 samples for each PT and MU average and the associated error bar

**Fig. 8:**
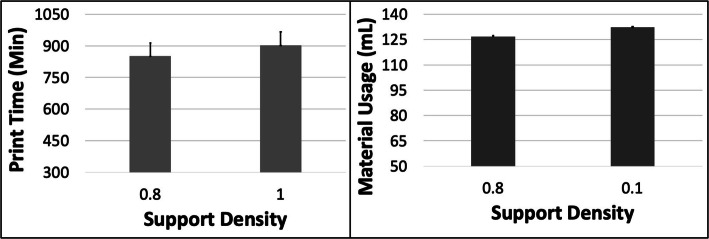
The response variables Print Time (PT) and Material Usage (MU) plotted against Support Density (SD) for the MICAB model. Sample size is 16 samples for each PT and MU average and the associated error bar

The PT and MU showed little change with ORN (< 3%) for the MICAB model. The PT reduced by ~ 44% with increased LH while the MU remained unchanged (Fig. 7). The PT and MU both increased with increasing SD, by ~ 4.2% and 5.9%, respectively (Fig. 8). The 5 most optimal (weighted) treatment combinations consist of the 0.1 mm LH and 0.80 SD with mostly smaller and larger ORNs (Table 5) as well as both levels of STS due to the minor effect of the latter 2 control variables.

**Table 5:** The 5 most weighted (W) optimal treatment combinations for the MICAB model

ORN (Deg)	LH (mm)	SD	STS (mm)	MU (mL)	PT (min)	W
15	0.1	0.8	0.5	125.6	612	−0.9320
0	0.1	0.8	0.4	126.88	606	−0.9309
0	0.1	0.8	0.5	126.43	611	−0.9224
30	0.1	0.8	0.5	126.34	612	−0.9207
30	0.1	0.8	0.4	127.03	609	−0.9194

### D – low grade glioma (LGG)

**Fig. 9:**
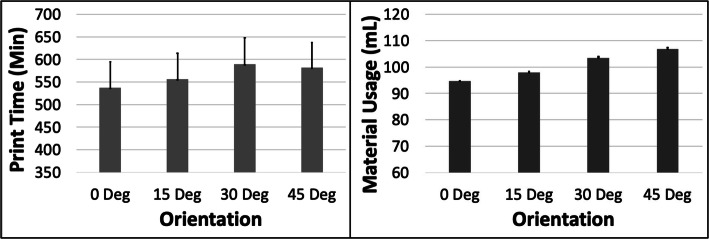
The response variables Print Time (PT) and Material Usage (MU) plotted against Orientation (ORN) for the LGG model. Sample size is 8 samples for each PT and MU average and the associated error bar

**Fig. 10:**
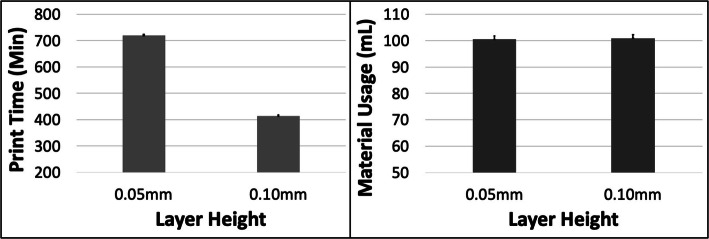
The response variables Print Time (PT) and Material Usage (MU) plotted against Layer Height (LH) for the LGG model. Sample size is 16 samples for each PT and MU average and the associated error bar

PT and MU both increased with ORN for the LGG model (Fig. 9). The maximum increase in PT was ~ 9.8% (between 0 and 30 deg) and in MU was ~ 12.9% (between 0 and 45 deg). The PT reduced by ~ 42.5% with increased LH but the MU remained unchanged (Fig. 10). An increase in SD had a minor impact on both PT and MU (< 2.5% increase). The 5 most optimal (weighted) treatment combinations consist of the 0.1 mm LH and smaller ORNs (Table 6) as well as both levels of STS and SD due to the minor effect of the latter 2 control variables.

**Table 6:** The 5 most weighted (W) optimal treatment combinations for the LGG model

ORN (Deg)	LH (mm)	SD	STS (mm)	MU (mL)	PT (min)	W
0	0.1	0.8	0.5	93.75	378	−1.0000
0	0.1	0.8	0.4	94.2	381	−0.9799
0	0.1	1	0.5	94.58	384	−0.9607
0	0.1	1	0.4	95.13	389	−0.9297
15	0.1	0.8	0.4	97.21	399	−0.8550

### E – renal cell carcinoma (RC)

**Fig. 11:**
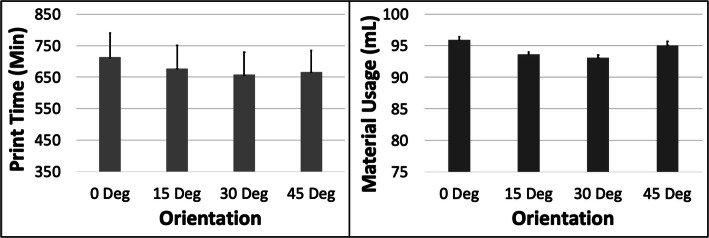
The response variables Print Time (PT) and Material Usage (MU) plotted against Orientation (ORN) for the RC model. Sample size is 8 samples for each PT and MU average and the associated error bar

**Fig. 12:**
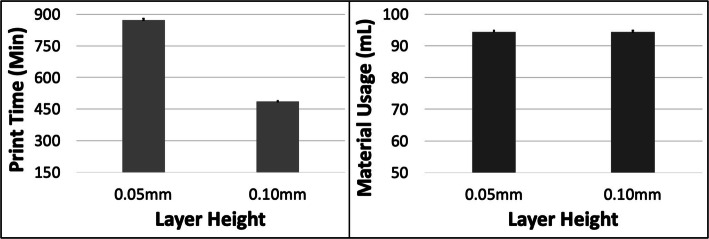
The response variables Print Time (PT) and Material Usage (MU) plotted against Layer Height (LH) for the RC model. Sample size is 16 samples for each PT and MU average and the associated error bar

The PT and MU both reduced with increasing ORN for the RC model (Fig. 11). The maximum reduction was ~ 7.7% in PT (between 0 and 30 deg) and ~ 2.9% in MU (between 0 and 30 deg). The PT reduced by ~ 44.4% with increasing LH while the MU remained constant (Fig. 12). An increase in SD resulted in a ~ 2% increase in both PT and MU. An increase in STS resulted in a ~ 1–1.5% reduction in both PT and MU. The 5 most optimal (weighted) treatment combinations consist of the 0.1 mm LH and larger ORNs (Table 7) as well as both levels of STS and SD due to the relatively minor effect of the latter 2 control variables.

**Table 7:** The 5 most weighted (W) optimal treatment combinations for the RC model

ORN (Deg)	LH (mm)	SD	STS (mm)	MU (mL)	PT (min)	W
30	0.1	0.8	0.5	91.13	461	−1.0000
30	0.1	0.8	0.4	92.09	466	−0.9510
45	0.1	0.8	0.5	92.49	467	−0.9348
30	0.1	1	0.5	93.13	470	−0.9034
15	0.1	1	0.5	92.85	477	−0.8854

### F – mandibular osteonecrosis (MO)

**Fig. 13:**
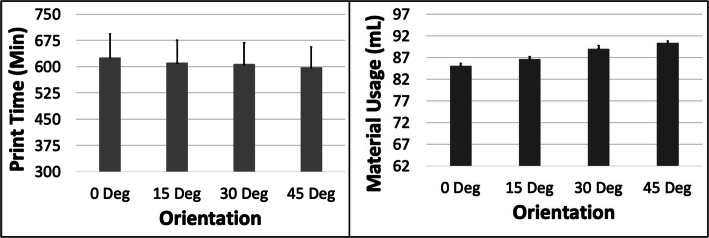
The response variables Print Time (PT) and Material Usage (MU) plotted against Orientation (ORN) for the MO model. Sample size is 8 samples for each PT and MU average and the associated error bar

**Fig. 14:**
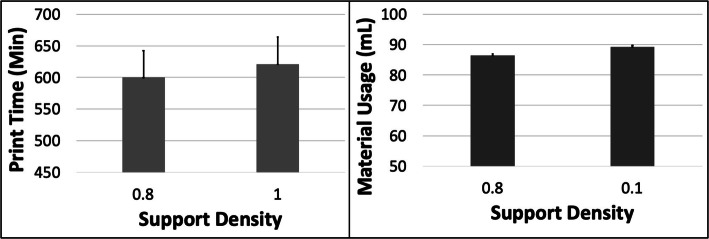
The response variables Print Time (PT) and Material Usage (MU) plotted against Support Density (SD) for the MO model. Sample size is 16 samples for each PT and MU average and the associated error bar

The PT reduced and MU increased with increasing ORN (Fig. 13). The maximum reduction in PT was ~ 4.6% (between 0 and 45 deg) and the maximum increase in MU was ~ 6.2% (between 0 and 45 deg) for the MO model. An increase in LH resulted in a reduction in PT of ~ 42.8% with constant MU. The PT increased ~ 3.5% and MU increased ~ 3.2% when the SD was increased (Fig. 14). The 5 most optimal (weighted) treatment combinations consist of the 0.1 mm LH, 0.8 SD and smaller ORNs (Table 8) with both levels of STS due to the relatively minor effect of the latter control variable.

**Table 8:** The 5 most weighted (W) optimal treatment combinations for the MO model

ORN (Deg)	LH (mm)	SD	STS (mm)	MU (mL)	PT (min)	W
0	0.1	0.8	0.5	83.24	435	−0.9617
15	0.1	0.8	0.5	84.95	428	−0.9574
30	0.1	0.8	0.5	86.66	430	−0.9113
0	0.1	0.8	0.4	84.78	444	−0.8868
15	0.1	0.8	0.4	86.19	438	−0.8843

### G – hepatic pseudoaneurysm (HPA)

**Fig. 15:**
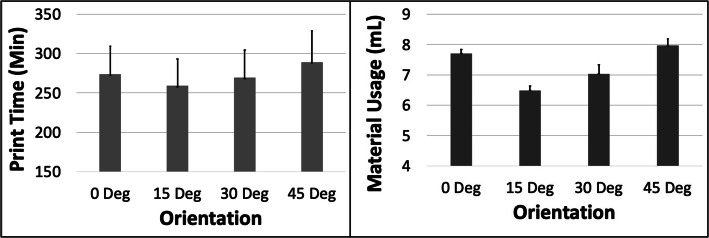
The response variables Print Time (PT) and Material Usage (MU) plotted against Orientation (ORN) for the HPA model. Sample size is 8 samples for each PT and MU average and the associated error bar

**Fig. 16:**
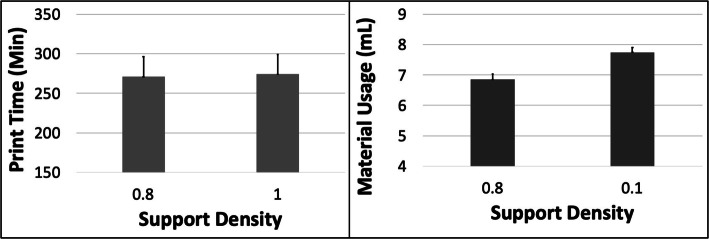
The response variables Print Time (PT) and Material Usage (MU) plotted against Support Density (SD) for the HPA model. Sample size is 16 samples for each PT and MU average and the associated error bar

The PT and MU both showed a complex pattern with respect to ORN (Fig. 15). The PT first reduced ~ 5.4% from 0 to 15 Deg ORN and subsequently increased by ~ 11.6% from 15 to 45 Deg ORN. The MU likewise reduced ~ 15.8% from 0 to 15 Deg ORN and later increased by ~ 22.7% from 15 to 45 Deg ORN. The PT reduced by ~ 52% with increased LH while MU reduced by ~ 2.5%. The MU increased ~ 13% with increased SD whereas PT increased ~ 1.2% (Fig. 16). The 5 most optimal (weighted) treatment combinations consist of the 0.1 mm LH, 0.8 SD and both smaller and larger ORNs (Table 9) with both levels of STS due to the relatively minor effect of the latter control variable.

**Table 9:** The 5 most weighted (W) optimal treatment combinations for the HPA model

ORN (Deg)	LH (mm)	SD	STS (mm)	MU (mL)	PT (min)	W
15	0.1	0.8	0.5	6.13	167	−0.9993
15	0.1	0.8	0.4	6.15	167	−0.9979
30	0.1	0.8	0.4	6.26	171	−0.9596
30	0.1	0.8	0.5	6.28	171	−0.9583
15	0.1	1	0.5	6.87	171	−0.9179

### H – basilar tip aneurysm (BTA)

**Fig. 17:**
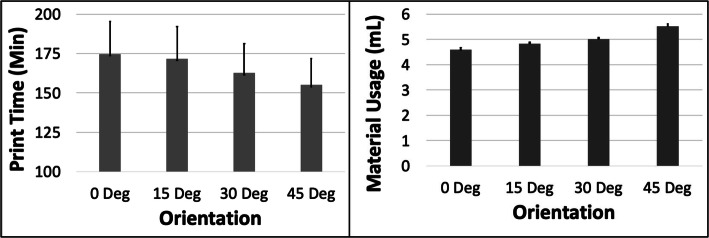
The response variables Print Time (PT) and Material Usage (MU) plotted against Orientation (ORN) for the BTA model. Sample size is 8 samples for each PT and MU average and the associated error bar

The PT reduced and MU increased with ORN (Fig. 17). The PT reduced by ~ 11.3% and MU increased by ~ 20.5% from 0 Deg to 45 Deg ORN. The PT reduced by ~ 47% with increased LH while MU increased by ~ 3.8%. The MU increased ~ 5.3% with increased SD whereas PT increased ~ 1.4%. The 5 most optimal (weighted) treatment combinations consist of the 0.1 mm LH, 0.8 SD and mostly larger ORNs (Table 10) with both levels of STS due to the relatively minor effect of the latter control variable.

**Table 10:** The 5 most weighted (W) optimal treatment combinations for the BTA model

ORN (Deg)	LH (mm)	SD	STS (mm)	MU (mL)	PT (min)	W
30	0.1	0.8	0.4	4.89	112	−0.8836
0	0.1	0.8	0.4	4.43	117	−0.8680
30	0.1	0.8	0.5	5.06	112	−0.8621
15	0.1	0.8	0.5	4.66	116	−0.8537
45	0.1	0.8	0.4	5.51	109	−0.8494

## Discussion

The material cost of 3D printing the model with largest MU is roughly ~US $20 (MICAB model) using the Formlabs VP material in our study which would be a minor fraction of the total cost for a 3D printing laboratory situated in a developed country. However, this could be a substantially larger fraction of the total cost in a developing country and therefore would warrant a larger weighting of MU in contrast to the 90–10 (PT-MU) optimization scheme employed in the present work. However, the methodology presented in this study could be easily adapted for such situations that demand a more tailored weighting of PT and MU based on the specific circumstance.

The Hollow models had more than twice the support volume fraction as the Solid models primarily due to the presence of support structures in the internal geometry of these models. MU varied relatively little compared to PT within each of the 8 models, which is because PT is determined by the laser toolpaths, post-layer peel operation and miscellaneous operations all of which depend on the number of layers (model Z height) and layer cross-sectional area that are in turn sensitive to all 4 control variables investigated in this study. All of the most optimal treatment combinations for each of the 8 models consisted of the 0.1 mm LH due to the reduced number of layers compared to the 0.05 mm LH. The reduction in PT was in the ~ 40–50% range with increased LH. In terms of ORNs both the smaller and larger levels were present in the optimal solutions since this control variable has a complex effect on PT and MU. A majority of the optimal solutions occurred at 0.8 SD due to the negative effect of this control variable on both PT and MU, albeit not as dominant as ORN or LH. Both levels of STS were present throughout the optimal treatment combinations due to the minor effect of this variable on PT and MU.

The Cube model showed a reduction in both PT and MU with increasing ORN due to a reduction in Z-height with increasing ORN (Fig. 1A). The 5 most optimal treatment combinations occurred at the 0.1 mm LH and 30/45 Deg ORNs, and both these values were at the higher end of the range for both control variables. However, it is important to note that the 45 Deg ORN for the Cube would result in a flat surface on top of the support pillars and likely lead to substantial warping and reduced print quality. The optimization scheme estimates that the 0.1 mm LH, 30 Deg ORN, 0.8 SD and 0.5 mm STS would result in optimized PT and MU for the Cube model.

For the LAA model the MU initially reduced and then increased with increasing ORN. This is due to the vertical alignment of the pulmonary veins from 0 to 15 Deg that resulted in fewer support pillars followed by increasing angular alignment from 15 to 45 Deg ORN (Fig. 1B). The PT and MU both increased with SD due to the Hollow nature of the LAA model. The 5 most optimal treatment combinations consisted of both smaller (15 Deg) due to the vertical alignment of the pulmonary veins, and larger (30 and 45 Deg) ORNs due to the reduced Z-height. The optimization scheme estimates that the 0.1 mm LH, 45 Deg ORN, 0.8 SD and 0.5 mm STS would result in optimized PT and MU for the LAA model.

For the MICAB model, the 5 most optimal treatment combinations consisted of both smaller (0 and 15 Deg) and larger (30 Deg) ORNs. This is due to the balance between fewer support pillars but larger Z-height in the lower ORNs and higher support pillar count but lower Z-height in the higher ORNs (Fig. 1C). The optimization scheme estimates that the 0.1 mm LH, 15 Deg ORN, 0.8 SD and 0.5 mm STS would result in optimized PT and MU for the MICAB model.

The PT and MU both increased with ORN for the LGG model which is due to the largest flat surface (bottom surface of the model) becoming more horizontally aligned and requiring a larger raft as well as more support pillars. Therefore, any reduction in PT due to the decrease in model Z-height with increasing ORN (Fig. 1D) was more than compensated for by an increase in support and raft volume (the opposite effect was observed in the Cube model). Four out of the 5 most optimal treatment combinations for the LGG model consisted of the 0 Deg (vertical) ORN. The optimization scheme estimates that the 0.1 mm LH, 0 Deg ORN, 0.8 SD and 0.5 mm STS would result in optimized PT and MU for the LGG model.

A similar balancing effect between reduced model Z-height and increased support volume with increasing ORN (Fig. 1E) was observed for the RC model as evidenced by the initially reducing PT and MU which subsequently increased with increasing ORN. Three out of the 5 most optimal treatment combinations consisted of the 30 Deg ORN, although both 0.8 and 1.0 SD levels were present. The optimization calculation estimates that the 0.1 mm LH, 30 Deg ORN, 0.8 SD and 0.5 mm STS would result in optimized PT and MU for the RC model.

The PT reduced whereas the MU increased with increasing ORN for the MO model due to the reducing Z-height and increasing support and raft volume (Fig. 1F). The slender nature of the MO model resulted in a noticeable increase in both PT and MU with increased SD. The optimization scheme estimates that the 0.1 mm LH, 0 Deg ORN, 0.8 SD and 0.5 mm STS would result in optimized PT and MU for the MO model.

The PT and MU both showed “U” shaped patterns with initially decreasing and later increasing values with increasing ORN for the HPA model. This is again because of the initial benefit from reducing model Z-height which is later more than compensated for by the increased support and raft volume (Fig. 1G). The differences between PT and MU values at various ORNs are larger compared to other models due to the relative dominance of support/raft volume fraction (> 52%). The optimization scheme estimates that the 0.1 mm LH, 15 Deg ORN, 0.8 SD and 0.5 mm STS would result in optimized PT and MU for the HPA model.

The PT decreased and MU increased with increasing ORN for the BTA model (Fig. 1H). The differences between PT and MU values at various ORNs were high similar to the HPA model due to the relative dominance of support/raft volume fraction (> 60%). The optimization scheme estimates that the 0.1 mm LH, 30 Deg ORN, 0.8 SD and 0.4 mm STS would result in optimized PT and MU for the BTA model.

The study has some limitations. First, this study presents a simulation-based approach for optimizing print time and material usage for 8 different medical anatomical models (7 medical and 1 reference cube) which ignores other aspects such as print surface quality, internal support structure removal, dimensional accuracy etc. Future research must factor such additional variables into the optimization scheme. Second, it is difficult to “standardize” orientations across different models; therefore, this study set the 0 Deg orientation as the vertical most orientation for each model. Third, the orientation was varied in only one degree of freedom and future studies should investigate additional degrees of freedom. Fourth, only models from single patients were used in the optimization framework and models between patients with the same conditions could vary substantially. Finally, the 90–10 weighting in the optimization scheme is subjective and in developing nations this skew would likely reduce due to availability of resources. However, the methodology presented can be adapted to concurrently optimize both print time and material usage based on the availability of resources and desired throughput for the 3D printing lab.

## Conclusions

The research successfully investigated the simulated effect of orientation, layer height, support density and tip size on print time and layer height for 7 different anatomical models and 1 reference cube. Hollow models demonstrated a higher proportion of support material due to internal supports. The optimization framework identified the 5 most optimal treatment combinations for each model using a 90–10 weighting for print time and material usage. The optimal solutions all included the 0.1 mm layer height since the goal of the optimization scheme was to minimize both print time and material usage. Orientation had a complex effect on both print time and material usage in certain models. Support density and tip size had a relatively minor impact on both response variables. The presented optimization framework could be adapted based on the individual circumstance of each 3D printing lab and/or to potentially incorporate additional response variables of interest.

## Data Availability

The raw dataset can be shared for non-commercial usage based on reasonable request.

## Abbreviations

VP: vat photopolymerization
LCD: liquid crystal display
DLP: digital light projection
HU: Hounsfield units
LAA: left atrial appendage
MICAB: minimally invasive coronary artery bypass
LGG: low grade glioma
RC: renal cell carcinoma
MO: mandibular osteonecrosis
HPA: hepatic pseudoaneurysm
BTA: basilar tip aneurysm
PT: print time
MU: material usage
ORN: orientation
LH: layer height
SD: support density
STS: support tip size
W: weight

## References

[1] Mitsouras D, Liacouras P, Imanzadeh A, Giannopoulos AA, Cai T, Kumamaru KK, George E, Wake N, Caterson EJ, Pomahac B, Ho VB, Grant GT, Rybicki FJ (2015). Medical 3D printing for the radiologist. Radiographics.

[2] Lichtenberger JP, Tatum PS, Gada S, Wyn M, Ho VB, Liacouras P (2018). Using 3D printing (additive manufacturing) to produce low-cost simulation models for medical training. Mil Med.

[3] Melchels FPW, Feijen J, Grijpma DW (2010). A review on stereolithography and its applications in biomedical engineering. Biomaterials.

[4] Ravi P, Chepelev L, Lawera N (2021). A systematic evaluation of medical 3D printing accuracy of multi-pathological anatomical models for surgical planning manufactured in elastic and rigid material using desktop inverted vat photopolymerization. Med Phys.

[5] Khodaygan S, Golmohammadi AH (2018). Multi-criteria optimization of the part build orientation (PBO) through a combined meta-modeling/NSGAII/TOPSIS method for additive manufacturing processes. Int J Interact Des Manuf.

[6] Jaiswal P, Patel J, Rai R (2018). Build orientation optimization for additive manufacturing of functionally graded material objects. Int J Adv Manuf Technol.

[7] Ravi P, Antoline S, Rybicki FJ. 3D printing of open-source respirators (including N95 respirators), surgical masks, and community mask designs to address COVID-19 shortages. In: 3D printing in medicine and its role in the COVID-19 pandemic: Springer; 2021. p. 91–106. 10.1007/978-3-030-61993-0_11.

[8] Griffiths CA, Howarth J, De Almeida-Rowbotham G (2016). A design of experiments approach for the optimisation of energy and waste during the production of parts manufactured by 3D printing. J Clean Prod.

[9] Jiang J, Ma Y. Path planning strategies to optimize accuracy, quality, build time and material use in additive manufacturing: a review. Micromachines. 2020;11(7). 10.3390/MI11070633. 10.3390/mi11070633 PMC7407298 32605325

[10] Jiang J, Xu X, Stringer J (2019). Optimization of process planning for reducing material waste in extrusion based additive manufacturing. Robot Comput Integr Manuf.

[11] Jiang J, Stringer J, Xu X (2019). Support optimization for flat features via path planning in additive manufacturing. 3D print. Addit Manuf.

[12] Kamio T, Hayashi K, Onda T, Takaki T, Shibahara T, Yakushiji T, Shibui T, Kato H (2018). Utilizing a low-cost desktop 3D printer to develop a “one-stop 3D printing lab” for oral and maxillofacial surgery and dentistry fields. 3D Print Med.

[13] Rubayo DD, Phasuk K, Vickery JM, Morton D, Lin WS. Influences of build angle on the accuracy, printing time, and material consumption of additively manufactured surgical templates. J Prosthet Dent. 2020:1–6. 10.1016/j.prosdent.2020.09.012. 10.1016/j.prosdent.2020.09.012 33143902

[14] Salmi M, Paloheimo KS, Tuomi J, Wolff J, Mäkitie A (2013). Accuracy of medical models made by additive manufacturing (rapid manufacturing). J Cranio-Maxillofacial Surg.

[15] Unkovskiy A, Bui PHB, Schille C, Geis-Gerstorfer J, Huettig F, Spintzyk S (2018). Objects build orientation, positioning, and curing influence dimensional accuracy and flexural properties of stereolithographically printed resin. Dent Mater.

[16] Hada T, Kanazawa M, Iwaki M, Arakida T, Soeda Y, Katheng A, Otake R, Minakuchi S (2020). Effect of printing direction on the accuracy of 3D-printed dentures using stereolithography technology. Materials (Basel).

[17] Tahayeri A, Morgan MC, Fugolin AP, Bompolaki D, Athirasala A, Pfeifer CS, Ferracane JL, Bertassoni LE (2018). 3D printed versus conventionally cured provisional crown and bridge dental materials. Dent Mater.

